# Preoperative prediction of clinical and pathological stages for patients with esophageal cancer using PET/CT radiomics

**DOI:** 10.1186/s13244-023-01528-0

**Published:** 2023-10-15

**Authors:** Xiyao Lei, Zhuo Cao, Yibo Wu, Jie Lin, Zhenhua Zhang, Juebin Jin, Yao Ai, Ji Zhang, Dexi Du, Zhifeng Tian, Congying Xie, Weiwei Yin, Xiance Jin

**Affiliations:** 1 Department of Radiation Oncology, Lishui Municipal Central Hospital, Lishui, 323000 China; 2 Department of Radiotherapy Center, 1st Affiliated Hospital of Wenzhou Medical University, Wenzhou, 325000 China; 3 Department of Respiratory, Lishui People’s Hospital, Lishui, 323000 China; 4 Department of Nuclear Medicine, 1st Affiliated Hospital of Wenzhou Medical University, Wenzhou, 325000 China; 5 Department of Radiology, 1st Affiliated Hospital of Wenzhou Medical University, Wenzhou, 325000 China; 6 Department of Medical and Radiation Oncology, 2nd Affiliated Hospital of Wenzhou Medical University, Wenzhou, 325000 China; 7 School of Basic Medical Science, Wenzhou Medical University, Wenzhou, 325000 China

**Keywords:** Esophageal neoplasms, PET-CT, Lymphatic metastasis, Neoplasm staging

## Abstract

**Background:**

Preoperative stratification is critical for the management of patients with esophageal cancer (EC). To investigate the feasibility and accuracy of PET-CT-based radiomics in preoperative prediction of clinical and pathological stages for patients with EC.

**Methods:**

Histologically confirmed 100 EC patients with preoperative PET-CT images were enrolled retrospectively and randomly divided into training and validation cohorts at a ratio of 7:3. The maximum relevance minimum redundancy (mRMR) was applied to select optimal radiomics features from PET, CT, and fused PET-CT images, respectively. Logistic regression (LR) was applied to classify the T stage (T_1,2_ vs. T_3,4_), lymph node metastasis (LNM) (LNM_(−)_ vs. LNM_(+)_), and pathological state (pstage) (I–II vs. III–IV) with features from CT (CT_LR_Score), PET (PET_LR_Score), fused PET/CT (Fused_LR_Score), and combined CT and PET features (CT + PET_LR_Score), respectively.

**Results:**

Seven, 10, and 7 CT features; 7, 8, and 7 PET features; and 3, 6, and 3 fused PET/CT features were selected using mRMR for the prediction of T stage, LNM, and pstage, respectively. The area under curves (AUCs) for T stage, LNM, and pstage prediction in the validation cohorts were 0.846, 0.756, 0.665, and 0.815; 0.769, 0.760, 0.665, and 0.824; and 0.727, 0.785, 0.689, and 0.837 for models of CT_LR_Score, PET_ LR_Score, Fused_ LR_Score, and CT + PET_ LR_Score, respectively.

**Conclusions:**

Accurate prediction ability was observed with combined PET and CT radiomics in the prediction of T stage, LNM, and pstage for EC patients.

**Critical relevance statement:**

PET/CT radiomics is feasible and promising to stratify stages for esophageal cancer preoperatively.

**Key points:**

• PET-CT radiomics achieved the best performance for Node and pathological stage prediction.

• CT radiomics achieved the best AUC for T stage prediction.

• PET-CT radiomics is feasible and promising to stratify stages for EC preoperatively.

**Graphical Abstract:**

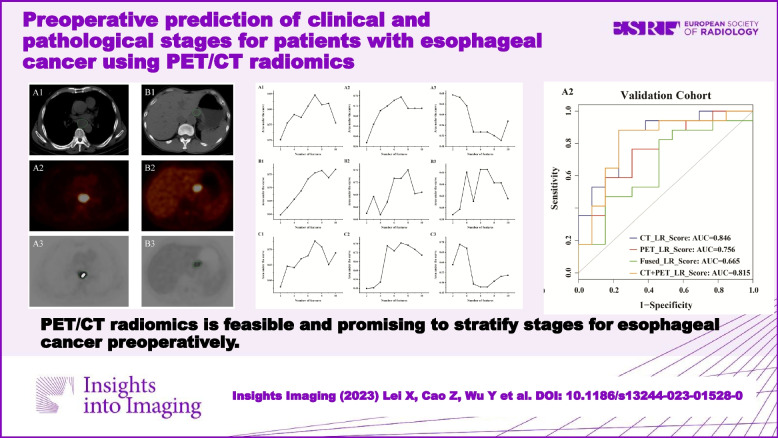

**Supplementary Information:**

The online version contains supplementary material available at 10.1186/s13244-023-01528-0.

## Introduction

The incidence of esophageal cancer (EC) is still increasing rapidly in the world [1]. Multidisciplinary treatment, such as combining surgery with neoadjuvant chemotherapy or chemoradiotherapy, offers the best chance of cure. However, the management of EC remains poor with an overall 5-year survival rate of around 5% to 20%, which turns EC into the eighth most common malignancy and the sixth leading cause of cancer-related mortality [2]. The management and prognosis of EC are determined by the level of tumor invasion and lymph node metastasis, which mainly relies on the American Joint Committee on Cancer (AJCC) tumor, node, and metastasis (TNM) staging classification [3]. Studies reported an overall 5-year survival rate of less than 20% as against 85% for patients with advanced-stage and early-stage, respectively [4]. The status of the stage is an important indicator for treatment decisions, as early-stage patients can be cured by surgery, and advanced-stage patients need chemotherapy combined with surgery [5]. Therefore, preoperative stratification of EC patients with corresponding TNM stage is critical for the management improvement of EC.

Currently, accurate T and N staging with endoscopic ultrasound (EUS) plays an important role to optimize treatment decisions for EC and has led to a mortality reduction and better recurrence-free survival rate [6]. However, studies demonstrated that EUS tends to overstate the depth of submucosal invasion which results in a low accuracy rate in early T staging, and it also performed poorly in N staging in comparison with T staging [7]. Computed tomography (CT), magnetic resonance imaging (MRI), and 18F-fluorodeoxyglucose positron emission tomography (FDG-PET) have been incorporated into the clinical practice to supplement the limitations of EUS [8]. However, despite the advances in imaging technologies and analysis over the past decades, the ability of accurate pretreatment staging for EC is still limited due to the low sensitivity and specificity profiles of each imaging modality [9].

The potential values of radiomics in predicting the TNM stages had been investigated intensively by extracting high-throughput quantitative features from images. Radiomics features from CT images were demonstrated to achieve an AUC from 0.637 to 0.857, and 0.728 to 0.840 in predicting the T stage and lymph node metastasis (LNM) [10–12], respectively. The role of MRI-based whole tumor histogram in the preoperative prediction of T staging and TNM were reported with an AUC of 0.773 and 0.762, respectively [13]. Although EUS and CT remain the most common image modality performed in the staging of EC, PET is also currently applied for the evaluation of clinical staging [14]. Studies demonstrated that the management of up to a third of EC patients could be changed by PET-CT [15]. Due to its quantitative nature, standardized uptake value (SUV) measures were usually utilized for EC diagnosis and staging [16]. Recently, PET-CT radiomics was applied to predict the clinical outcomes for patients with locally advanced EC who underwent chemoradiotherapy and achieved reasonable accuracy [17].

Pathological staging is usually a combination of clinical stage with surgical results and is generally a more precise way to find out how far EC cancer spread [18]. However, due to the marked survival differences demonstrated by the data analysis in the eighth edition of AJCC staging, simple sharing of stage groups among classifications is not possible [19]. Preoperative prediction for the pathological stage (pstage) is of great clinical value for decision-making. The purpose of this study is to investigate the potential and accuracy of radiomics features extracted from CT, PET, and fused PET-CT images in the preoperative prediction of clinical and pathological stages for EC patients.

## Materials and methods

### Patients

Patients diagnosed with EC from January 2011 to December 2020 were analyzed in this retrospective study through searching electronic medical records in the author’s hospital. All the enrolled patients were pathologically proved EC, and an 18F-FDG PET-CT examination was performed within one month before operation, without radiotherapy and chemotherapy before operation. TNM stages were obtained by a senior radiation oncologist with over 15 years of experience according to the AJCC eighth edition TNM staging guidelines for esophageal cancer with postoperative pathology results [19]. Patients without preoperative PET-CT examination, missing PET-CT images, incomplete pathological information, non-SCC pathological type, and treatment other than surgery were excluded. The Ethics Committee in Clinical Research of the author's hospital approved this study (ECCR# 2,019,059) as it was conducted according to the Helsinki Declaration.

### FDG PET-CT acquisition

PET/CT images were acquired on a Philips PET-CT scanner (Gemini TF 64 w/ TOF Performance, the Netherlands). A 3.7 MBq/kg of 18F-FDG was administrated intravenously for each patient. Approximately one hour after the tracer injection, CT images were acquired with a voltage of 120 kV, a tube current of 300 mA, a section thickness of 2.5 mm, and a matrix of 512 × 512. The PET images were scanned with a scanning layer thickness of 4 mm and a matrix of 144 × 144. After imaging was completed, all data were transferred to Philips Post-processing Workstation (EBW 3.0) for reconstruction to obtain PET, CT, and PET-CT fusion image. The fused PET-CT image in this study was obtained by importing the PET image and CT image into the external software Accucontour 3.2 (Manteia Corp, Xiamen, China, www.manteiatech.com) for 1:1 fusion.

### Tumor segmentation

**Fig. 1:**
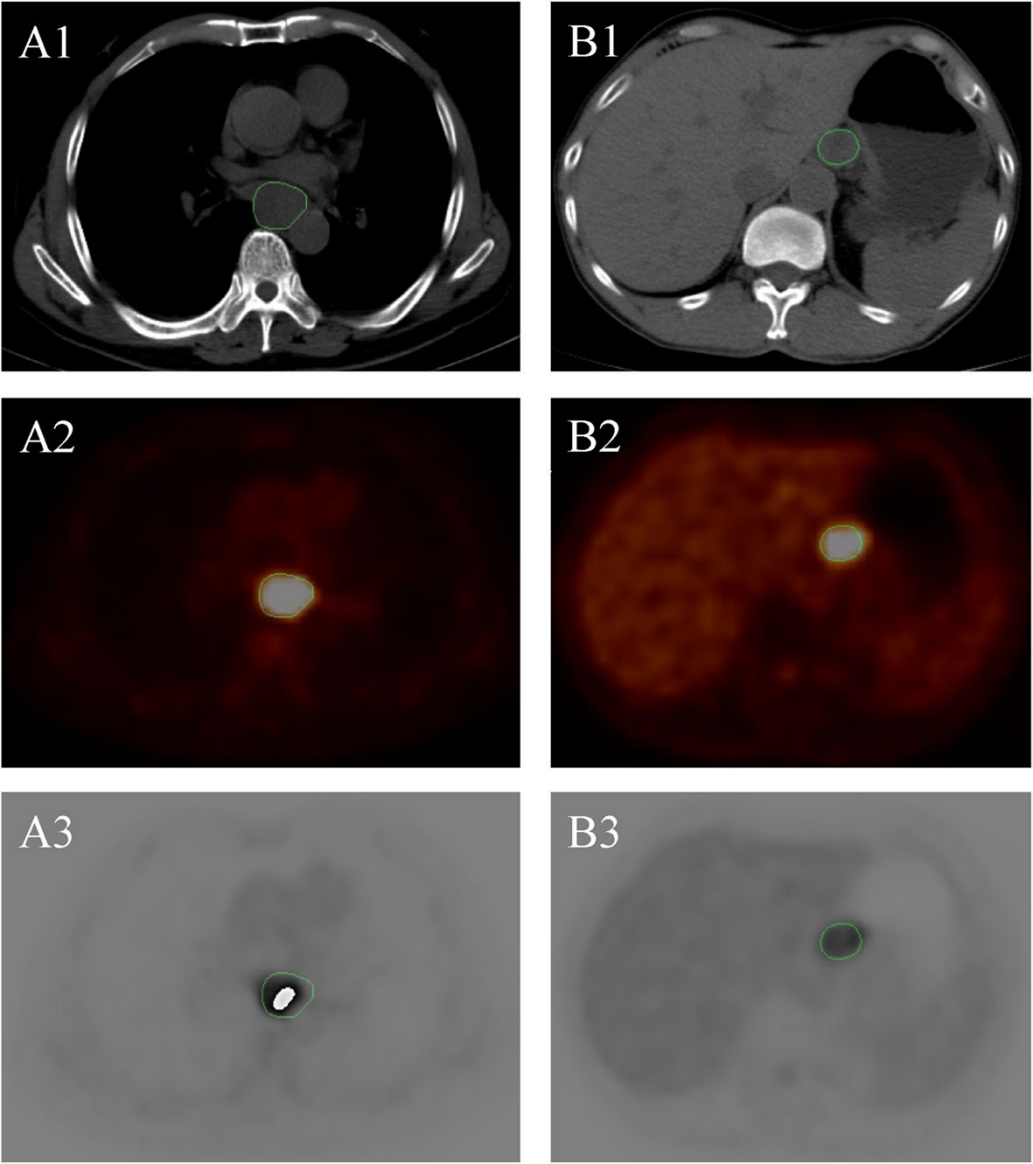
Demonstration of typical target segmentation of esophageal cancer. Forty percent threshold of 76-year-old man ESCC patient’s SUVmax: **A1** CT image; **A2** PET image; **A3** fused CT and PET image. Threshold of 65-year-old man ESCC patient’s SUVmax: **B1** CT image; **B2** PET image; **B3** fused CT and PET image

The PET-CT images were fused by Lifex software (version 6.3, Orsay, France, https://www.lifexsoft.org) [20]. A junior radiation oncologist manually delineated each layer of the tumor region in the CT image and then mapped it into the PET image and fused PET-CT image for further analysis. For PET images with a high SUVmax value (40%SUVmax >  = 3), the 40% threshold of SUVmax was used to manually determine the edge of the primary tumor. For PET images with a low SUVmax value (< 3), the value of SUVmax was used to manually determine the edge of the primary tumor. A senior radiation oncologist with over 15 years of experience was consulted for the final verification and approval of the contours. A typical contour of target volumes on PET and CT images is presented in Fig. 1.

### Image preprocessing and radiomics feature extraction

PyRadiomics (Python package, https://www.python.org) was applied to extract radiomics features from contoured target volumes in the CT, PET, and fused PET-CT images, respectively [21]. The CT, PET, and fused PET-CT images were normalized using the configuration files provided with Pyradomics with the voxel size resampled to 1.5 × 1.5 × 1.5, and the bin width of pixel level set to 16. Features according to the Image Biomarker Standardization Initiative (IBSI) were extracted: first-order histogram statistics, gray-level co-occurrence matrix (GLCM), gray-level size zone matrix (GLSZM), gray-level run-length matrix (GLRLM), gray-level different matrix (GLDM), neighborhood gray-tone difference matrix (NGTDM) [22].

### Radiomics features screening and model building

The enrolled patients were randomly divided into training and validation cohorts at a ratio of 7:3. In the training cohort, the maximum relevance minimum redundancy (mRMR) was used to rank the features according to their predictive ability to select the optimal features for radiomics model building. To avoid overfitting, the top 2–10 features were selected following the principle that the maximum number of features was 1/10 of the number of patients. Logistic regression (LR) models were built in the training cohort based on the selected radiomic features. The optimal number of features for radiomics model construction was determined by the AUC value generated by the logistic regression model. The prediction probability of models is evaluated using the LR radiomics score (LR_Score). Four models were established and named as CT_LR_Score, PET_LR_Score, Fused_LR_Score, CT LR_Score + PET_LR_Score (CT + PET_LR_Score), according to the optimal features from CT images, PET images, fused PET-CT images, respectively. The receiver operating characteristic (ROC) curve, accuracy, sensitivity, and specificity were used to evaluate the predictive performance of these radiomics models.

### Statistical analysis

Statistical analysis was conducted by R studio (version 4.0.4, Vienna, Austria, http://www.Rproject.org) in this study with LR in the “glmnet” package, mRMR in the “mRMRe” package, and “pROC” package was used for ROC curves. Univariate and multivariate regression analysis to evaluate the predictive value of clinical parameters by SSPS software (version 19.0, IBM Corp, Armonk, USA). A *p* < 0.05 was considered statistically significant.

## Results

**Fig. 2:**
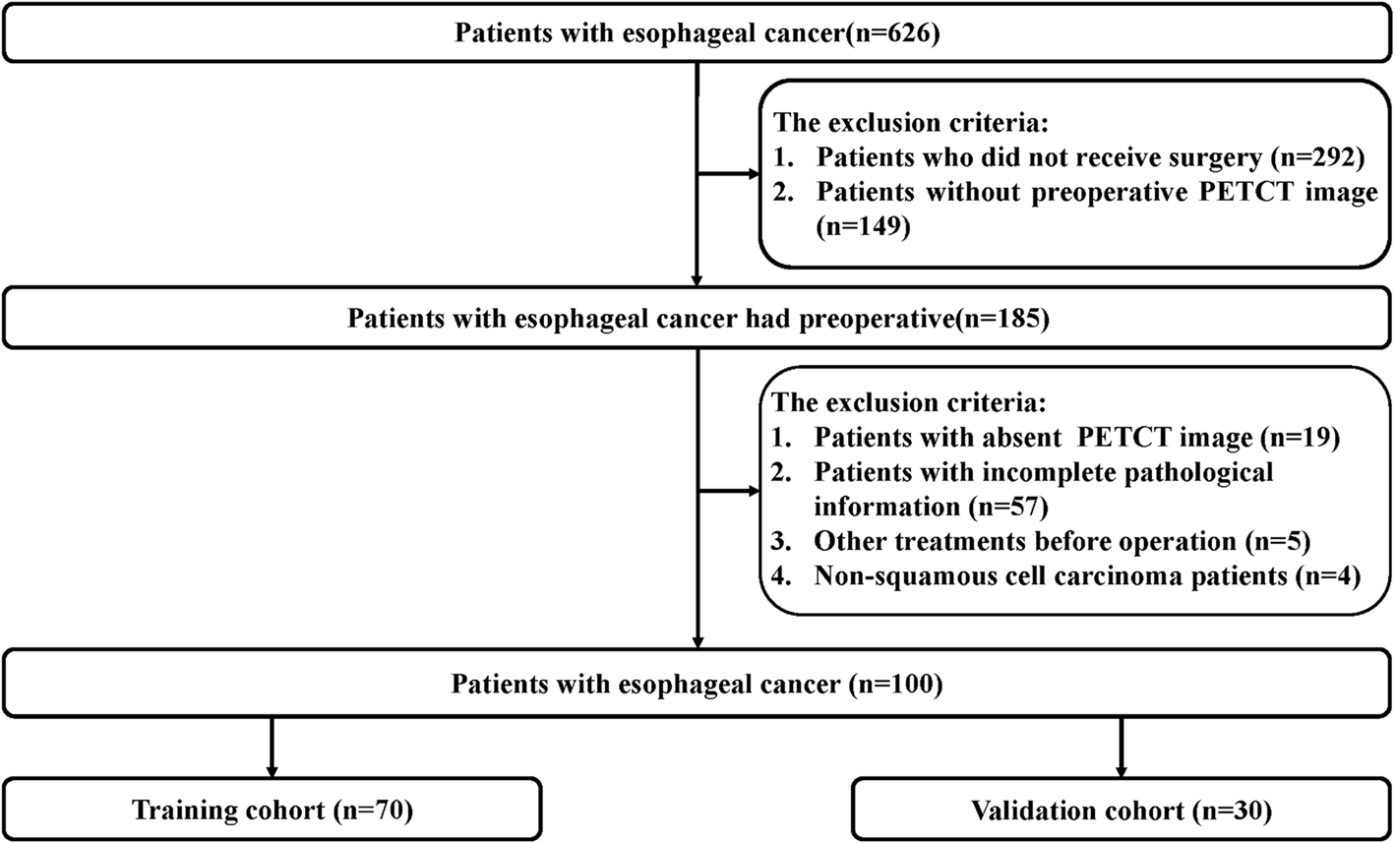
The flowchart of patients’ enrollment

A total of 626 patients diagnosed with EC from January 2011 to December 2020 were reviewed. As shown the flowchart for patient selection in Fig. 2, a total of 100 patients with squamous cell carcinoma (SCC) were included in the study for final analysis with a mean age of 65.5 years old (range from 45 to 85 years old), which included 48 patients with T_1,2_ and 52 patients with T_3,4_, 56 patients with LNM ( +) and 44 patients with LNM ( −), and 57 patients with pathological stage (pstage) I–II and 43 patients with pstage III–IV, respectively. Detailed characteristics of these patients are presented in Table 1.

**Table 1:** Clinicopathological characteristics of enrolled patients

Variables	Total
Gender	
Male	88
Female	12
Age	
Range	45–85
Mean ± SD	65.50 ± 8.71
Pathological T stage	
T_1–2_	48
T_3–4_	52
Pathological N stage	
N_0_	56
N_1–3_	44
Pathological M stage	
M0	78
M1	22
Pathological stage	
I–II	57
III–IV	43
Tumor length (cm)	
< 5	76
≥ 5	24
Tumor grade	
Low	35
Middle	47
High	18
Tumor location	
Upper	5
Middle	51
Lower	44

**Fig. 3:**
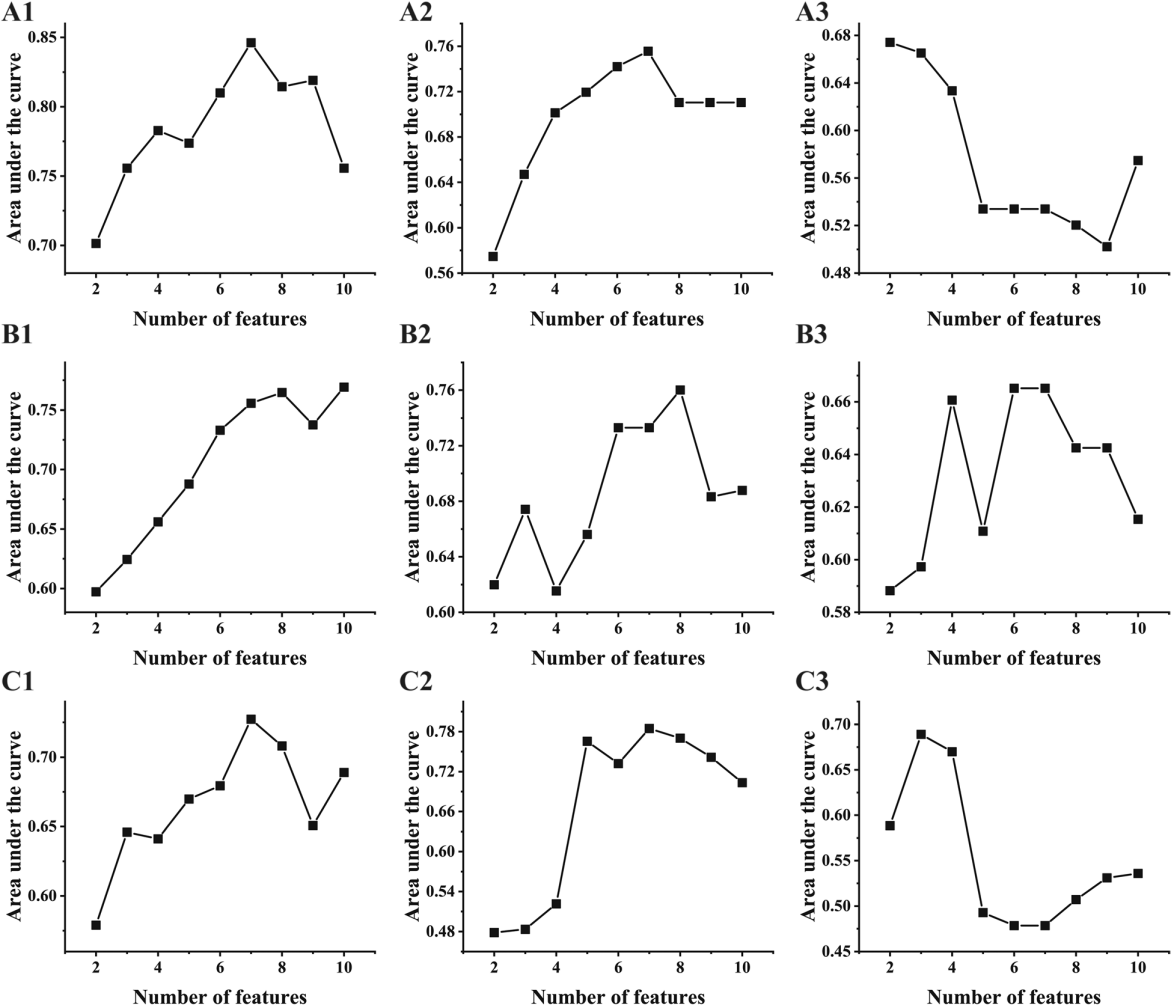
Feature selection using the mRMR method. **A1–3** Screened from CT, PET, and fused radiomics features for T stage, respectively. **B1–3** Screened from CT, PET, and fused radiomics features for LNM, respectively. **C1–3** Screened from CT, PET, and fused for pstage, respectively

A total of 1288 features were extracted from each imaging modality. During the optimal feature selection with mRMR, the principle that the maximum number of features is 1/10 of patients was followed [23]. The number of optimal features under no overfitting condition was determined by the corresponding largest area under curve (AUC) value generated by LR. As shown in Fig. 3, there were 7, 7, and 3 features were selected for T stage classification, 10, 8, and 6 features selected for LNM classification, and 7, 7, and 3 features selected for pstage I–II and pstage III–IV classification from CT radiomics features, PET radiomics features, and fused PET-CT radiomics features, respectively. Detailed features were presented in Supplemental Table S1.

**Fig. 4:**
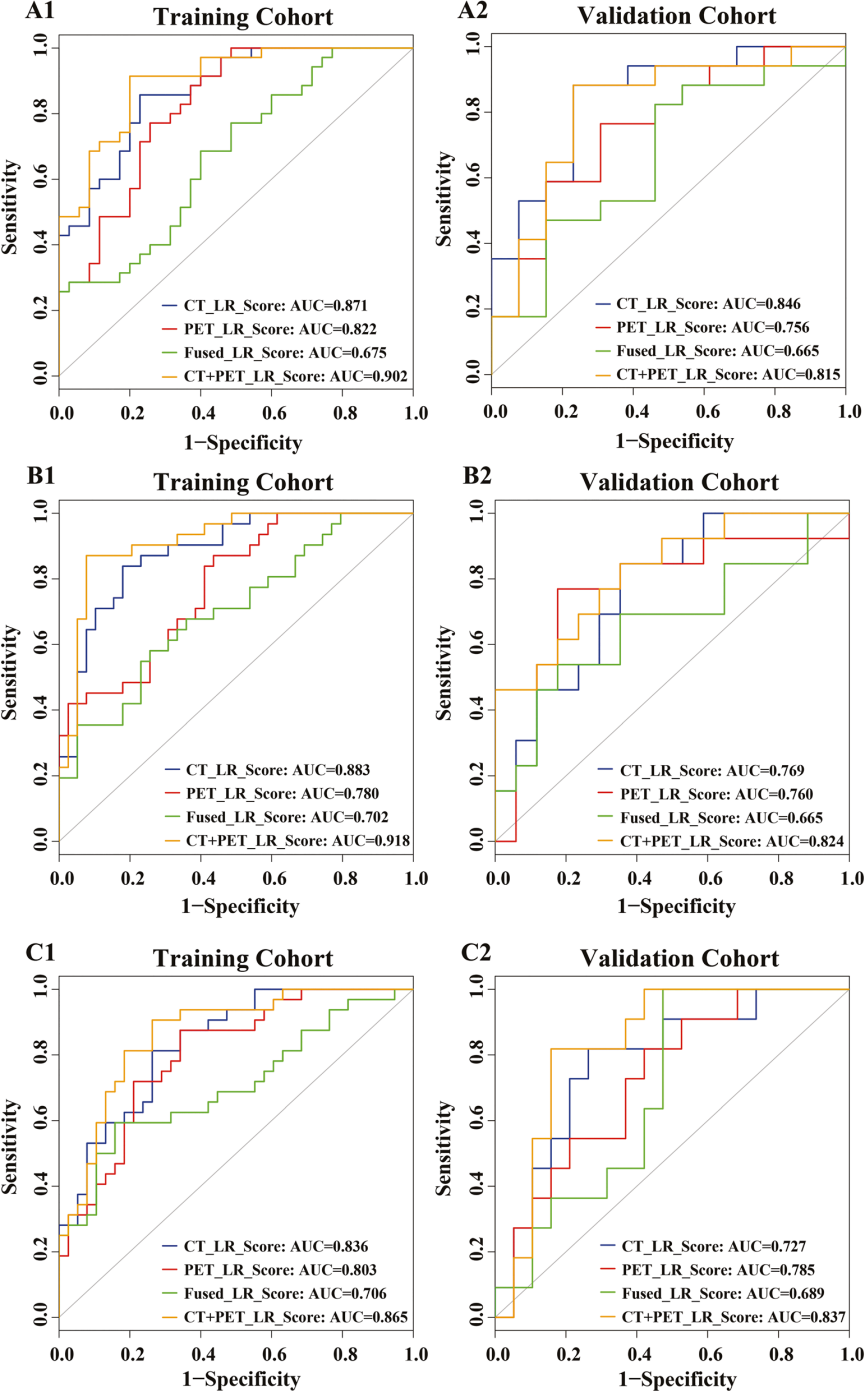
The performance evaluation with receiver operation curves for four logistic regression models. **A1, 2** is the ROC curve for predicting T stage in the training cohort and validation cohort, respectively. **B1, 2** is the ROC curve for predicting LNM in the training cohort and validation cohort, respectively. **C1, 2** is the ROC curve for predicting pstage in the training cohort and validation cohort, respectively

Five clinical parameters (gender, age, tumor length, tumor grade, tumor location) were not significantly correlated with T stage, LNM, and pstage according to univariate analysis (*p* < 0.05), as shown in Supplemental Table S2. The ROCs for the evaluation of the performance of the constructed radiomics models in the training and validation cohorts are shown in Fig. 4. The AUC values for T stage, LNM, and pstage prediction in the validation cohorts were 0.846, 0.756, 0.665, and 0.815; 0.769, 0.760, 0.665, and 0.824; and 0.727, 0.785, 0.689, and 0.837 for models of CT_LR_Score, PET_ LR_Score, Fused_ LR_Score, and CT + PET_ LR_Score, respectively. A detailed performance of these four models is presented in Table 2.

**Table 2:** The performance of the T stage, N stage, and pstage model for the training and validation cohorts

Stage	Model	Training cohort				Validation cohort			
		**AUC (95% CI)**	**Accuracy**	**Specificity**	**Sensitivity**	**AUC (95% CI)**	**Accuracy**	**Specificity**	**Sensitivity**
T stage	CT_LR_Score	0.871 (0.792–0.951)	0.814	0.771	0.857	0.846 (0.702–0.991)	0.833	0.769	0.882
	PET_LR_Score	0.822 (0.725–0.920)	0.757	0.743	0.771	0.756 (0.576–0.936)	0.733	0.692	0.765
	Fused_LR_Score	0.675 (0.549–0.801)	0.643	0.600	0.686	0.665 (0.460–0.870)	0.700	0.538	0.824
	CT + PET_LR_Score	0.902 (0.834–0.971)	0.857	0.800	0.914	0.815 (0.646–0.983)	0.833	0.769	0.882
N stage	CT_LR_Score	0.883 (0.806–0.961)	0.829	0.821	0.839	0.769 (0.600–0.939)	0.733	0.647	0.846
	PET_LR_Score	0.780 (0.674–0.886)	0.700	0.564	0.871	0.760 (0.566–0.954)	0.867	0.824	0.769
	Fused_LR_Score	0.702 (0.579–0.825)	0.671	0.744	0.581	0.665 (0.456–0.874)	0.700	0.824	0.538
	CT + PET _LR_Score	0.918 (0.852–0.985)	0.960	0.923	0.871	0.824 (0.674–0.973)	0.733	0.647	0.846
pstage	CT_LR_Score	0.836 (0.745–0.927)	0.771	0.737	0.812	0.727 (0.542–0.912)	0.667	0.579	0.818
	PET_LR_Score	0.803 (0.701–0.904)	0.757	0.658	0.875	0.785 (0.611–0.958)	0.767	0.737	0.818
	Fused_LR_Score	0.706 (0.580–0.831)	0.729	0.842	0.594	0.689 (0.496–0.882)	0.700	0.526	1.000
	CT + PET _LR_Score	0.865 (0.780–0.950)	0.814	0.737	0.906	0.837 (0.689–0.986)	0.833	0.842	0.818

*AUC* area under the curve

## Discussion

Accurate clinical staging is one of the most important prognostic factors for the management of EC. The feasibility and accuracy of radiomics features extracted from CT, PET, and fused PET-CT images in the preoperative prediction of stages for EC patients were investigated with LR. Radiomics models combining CT and PET radiomics features (CT + PET_ LR_Score) achieved a best performance for N stage and pstage prediction with an AUC of 0.824 and 0.837 in the validation cohort, respectively. CT radiomics model (CT_LR_Score) demonstrated an AUC of 0.871 and 0.846 for T stage prediction in the training and validation cohorts, respectively.

Despite tremendous advances in imaging over the past few years, detecting the clinical staging status with a single noninvasive imaging modality is still limited and continues to be a source of frustration for radiologists [24]. Currently, although CT is widely applied for nodal staging in patients with EC, a relatively low sensitivity was reported with the accuracy, sensitivity, and specificity of 54.5%, 39.7%, and 77.3%, respectively [25]. False-positive interpretations for enlarged nodes and misses of the micrometastases in small nodes were also criticized for CT [26]. In this study, accuracy, sensitivity, and specificity of 0.733, 0.647, and 0.846 were observed in the validation cohorts with CT radiomics alone for LNM prediction of EC, respectively. The AUC of CT alone radiomics is 0.769 in the validation cohort, which is close to the reported 0.771 and 0.773 AUC in the studies of Shen et al. and Tan et al. for LNM prediction for EC [11, 12]. As the feature dimension increases, the difficulty of classification will increase, which may lead to the accuracy of the combined CT and PET model for predicting LNM being lower than that of the PET model alone. In order to maximize the correlation between features and classification variables, and minimize the correlation between features, mRMR was applied in this study for optimal feature selection [27].LR was applied due to its advantage in training speed and interpretability in model construction [11].

EUS is widely applied for T staging in patients with EC. However, the accuracy of EUS in the assessment of tumor invasion depth and T stage was frequently affected by the esophageal stenosis [28]. A low accuracy of 58.6% was reported previously for EUS in the prediction of T stage for EC [29].In the study, radiomics models with CT radiomics (CT_LR_Score) and combined PET and CT radiomics (CT + PET_ LR_Score) all achieved an accuracy of 83.3% in the prediction of T stage for EC. However, both the AUC values of CT_LR_Score (0.846) and CT + PET_ LR_Score (0.815) were inferior to the reported 0.857 in the study of Yang et al. using CT radiomics signature with 116 EC patients for T stage prediction [10].

PET-CT is also frequently applied to determine the stage status non-invasively for patients with EC using parameters of maximum SUV (SUVmax), mean SUV, metabolic tumor volume, total lesion glycolysis, and intratumoral metabolic heterogeneity, among others [30, 31]. Studies have shown that combined with PET-CT and parameter SUVmax, the prediction accuracy reached 73.3% and 82%, indicating that PET-CT is helpful in the prediction of T staging of esophageal cancer [32, 33]. Wang et al. and Lee et al. found that the accuracy of PET/MRI in predicting the stage of primary tumors reached 85.7% and 83.3%, which was consistent with the diagnostic model of this study [33, 34]. Recently, Jayaprakasam et al. on PET-CT radiomics predicting stage in patients with locally advanced ESCC has been reported. The study found that radiomics models based on CT, PET, and PET-CT performed well in predicting tumor and N category with diagnostic accuracy of more than 70% [17]. In this study, for the prediction of T stage, the LR model demonstrated better performance of CT alone radiomics in comparison with combined CT and PET radiomics and PET alone radiomics. The diagnostic accuracy of the three models was higher than that of Jayaprakasam et al. (ACC > 73%), but the AUC was still slightly lower (0.815 vs.0.900) in the validation cohort. For the prediction of the LNM category, the combined CT and PET radiomics improved the prediction performance compared with CT_LR_Score and PET_LR_Score, which is consistent with the results of Jayaprakasam et al. The radiomics features extracted from fused PET-CT images have the lowest performance for T staging, LNM, and pstage, which may be due to the loss of important information of tumor region in images fused by external software, thus reducing the performance of the model.

Nearly half the patients we enrolled were pathologically diagnosed with advanced esophageal cancer (pstage III–IV), but all patients were treated directly with surgery, without preoperative chemoradiotherapy, which is an important limitation in the clinical application of EC patients. Another limitation of this study is that clinical risk factors of the stage were not investigated together with radiomics features, especially integrating with PET radiomics, which has not been investigated previously. Finally, a relatively small number of EC patients were enrolled in this study from a single center. Further studies with more cases and external validation cohorts are necessary.

## Conclusion

Accurate prediction ability was observed with combined PET and CT radiomics in the prediction of T stage, LNM, and pstage for EC patients in this study. PET-CT-based radiomics is promising to improve the diagnosis and management for patients with EC.

### Supplementary Information

**Additional file 1:** **Table S1.** Selected radiomics features from CT, PET and fused PET/CT for T stage, N stage and pstage.** Table S2.** Univariate logistic regression analysis of clinical parameters at three stages in training cohort.

## Data Availability

The datasets generated and analyzed during the current study are not publicly available but are available from the corresponding author on reasonable request.

## Abbreviations

AUC: Area under the curve
EC: Esophageal cancer
ECCR: Ethics Committee in Clinical Research
ESCC: Esophageal squamous cell carcinoma
GLCM: Gray-level co-occurrence matrix
GLDM: Gray-level different matrix
GLRLM: Gray-level run-length matrix
GLSZM: Gray-level size zone matrix
LNM: Lymph node metastasis
LR_Score: Logistic regression radiomics score
mRMR: Maximum relevance minimum redundancy
pstage: Pathological stages
ROC: Receiver operating characteristic
