# Supplementary material for: Preoperative prediction of clinical and pathological stages for patients with esophageal cancer using PET/CT radiomics
Source: Insights Imaging. 2023 Oct 15;14:174. doi: 10.1186/s13244-023-01528-0 (PMC10577114; doi:10.1186/s13244-023-01528-0)
Supplement: Supplementary file 1 — Additional file 1: Table S1. Selected radiomics features from CT, PET and fused PET/CT for T stage, N stage and pstage. Table S2. Univariate logistic regression analysis of clinical parameters at three stages in training cohort. [file 13244_2023_1528_MOESM1_ESM.docx]

| **Table S1** Selected radiomics features from CT, PET and fused PET/CT for T stage, N stage and pstage | |
| --- | --- |
| **Dataset** | **Features** |
| **T stage** | |
|  | wavelet-HLL_firstorder_10Percentile |
|  | wavelet-LHH_firstorder_Skewness |
|  | log-sigma-2-0-mm-3D_firstorder_Skewness |
| CT | original_gldm_DependenceVariance |
|  | wavelet-HHL_gldm_SmallDependenceLowGrayLevelEmphasis |
|  | wavelet-LHH_glszm_SizeZoneNonUniformityNormalized |
|  | wavelet-LHL_glcm_InverseVariance |
|  | |
|  | wavelet-HHH_firstorder_Skewness |
|  | wavelet-HHL_firstorder_Kurtosis |
|  | log-sigma-3-0-mm-3D_glcm_Imc1 |
| PET | wavelet-HHH_glszm_SmallAreaEmphasis |
|  | original_shape_Flatness |
|  | wavelet-LLH_firstorder_Skewness |
|  | wavelet-HLH_glcm_JointEnergy |
|  | |
|  | wavelet-LHL_firstorder_Kurtosis |
| Fused | wavelet-HHH_glrlm_RunVariance |
|  | log-sigma-3-0-mm-3D_glszm_SmallAreaLowGrayLevelEmphasis |
|  | |
| **N stage** | |
|  | wavelet-LLH_glszm_LowGrayLevelZoneEmphasis |
|  | original_ngtdm_Busyness |
|  | original_firstorder_Median |
|  | log-sigma-4-0-mm-3D_glcm_Imc1 |
| CT | wavelet-HLH_firstorder_Median |
|  | wavelet-LHL_glcm_ClusterShade |
|  | wavelet-HHL_firstorder_Median |
|  | log-sigma-1-0-mm-3D_glszm_GrayLevelNonUniformityNormalized |
|  | log-sigma-1-0-mm-3D_glszm_LowGrayLevelZoneEmphasis |
|  | log-sigma-4-0-mm-3D_glszm_SmallAreaLowGrayLevelEmphasis |
|  | |
|  | wavelet-HLL_glcm_ClusterShade |
|  | wavelet-LLH_gldm_LargeDependenceLowGrayLevelEmphasis |
|  | wavelet-HHH_glszm_GrayLevelVariance |
| PET | wavelet-HHL_glszm_SmallAreaLowGrayLevelEmphasis |
|  | wavelet-HLH_glszm_SmallAreaEmphasis |
|  | wavelet-LLH_firstorder_Kurtosis |
|  | wavelet-HLH_ngtdm_Complexity |
|  | wavelet-LHH_gldm_DependenceNonUniformityNormalized |
|  | |
|  | log-sigma-3-0-mm-3D_glszm_LargeAreaLowGrayLevelEmphasis |
|  | wavelet-HLL_glszm_SmallAreaLowGrayLevelEmphasis |
| Fused | wavelet-HLH_glcm_ClusterShade |
|  | wavelet-LLH_firstorder_Kurtosis |
|  | log-sigma-5-0-mm-3D_firstorder_90Percentile |
|  | log-sigma-2-0-mm-3D_gldm_DependenceVariance |
|  | |
| **pstage** | |
|  | wavelet-HHL_glszm_SmallAreaEmphasis |
|  | wavelet-HHH_glszm_GrayLevelVariance |
|  | log-sigma-1-0-mm-3D_glszm_SmallAreaLowGrayLevelEmphasis |
| CT | wavelet-LHL_glszm_SmallAreaEmphasis |
|  | wavelet-HLH_firstorder_Median |
|  | wavelet-HLL_firstorder_Skewness |
|  | log-sigma-4-0-mm-3D_glszm_SmallAreaLowGrayLevelEmphasis |
|  | |
|  | wavelet-HHH_glszm_GrayLevelNonUniformityNormalized |
|  | wavelet-HHL_glszm_SmallAreaLowGrayLevelEmphasis |
|  | log-sigma-2-0-mm-3D_glszm_LargeAreaHighGrayLevelEmphasis |
| PET | wavelet-HLH_ngtdm_Contrast |
|  | wavelet-LHH_gldm_DependenceNonUniformityNormalized |
|  | wavelet-HHH_glszm_SizeZoneNonUniformityNormalized |
|  | wavelet-LLH_gldm_LargeDependenceLowGrayLevelEmphasis |
|  | |
|  | log-sigma-5-0-mm-3D_glszm_LargeAreaHighGrayLevelEmphasis |
| Fused | original_shape_MajorAxisLength |
|  | wavelet-LHH_firstorder_Mean |

| **Table S2** Univariate logistic regression analysis of clinical parameters at three stages in training cohort | | | | | | | |
| --- | --- | --- | --- | --- | --- | --- | --- |
| **Factors** | **Univariate logistic regression** | | | | | | |
|  | **OR (95% CI)** | | |  | **P value** | | |
|  | **T stage** | **N stage** | **pStage** |  | **T stage** | **N stage** | **pStage** |
| Gender | 2.207 (0.505-9.639) | 4.412 (0.488-39.914) | 1.137 (0.235-5.504) |  | 0.293 | 0.187 | 0.873 |
| Age | 0.976 (0.922-1.034) | 0.985 (0.933-1.040) | 0.972 (0.922-1.025) |  | 0.406 | 0.595 | 0.295 |
| Tumor length | 0.568 (0.198-1.628) | 0.631 (0.210-1.891) | 0.497 (0.164-1.507) |  | 0.292 | 0.411 | 0.217 |
| Tumor location | 0.428 (0.170-1.080) | 1.028 (0.451-2.342) | 1.303 (0.580-2.926) |  | 0.073 | 0.947 | 0.521 |
| Tumor grade | 1.553 (0.766-3.148) | 0.862 (0.446-1.666) | 0.764 (0.403-1.445) |  | 0.222 | 0.659 | 0.407 |
| pStage, pathological stage | | | | | | | |
